# Chip-Oriented Fluorimeter Design and Detection System Development for DNA Quantification in Nano-Liter Volumes

**DOI:** 10.3390/s100100146

**Published:** 2009-12-28

**Authors:** Da-Sheng Lee, Ming-Hui Chen

**Affiliations:** Department of Energy and Refrigerating Air-Conditioning Engineering, National Taipei University of Technology, Taipei 10608, Taiwan; E-Mail: Smartlab610@gmail.com

**Keywords:** polymerase chain reaction (PCR), DNA quantification, chip-oriented fluorimeter design, real-time PCR on a chip system, S/N ratio

## Abstract

The chip-based polymerase chain reaction (PCR) system has been developed in recent years to achieve DNA quantification. Using a microstructure and miniature chip, the volume consumption for a PCR can be reduced to a nano-liter. With high speed cycling and a low reaction volume, the time consumption of one PCR cycle performed on a chip can be reduced. However, most of the presented prototypes employ commercial fluorimeters which are not optimized for fluorescence detection of such a small quantity sample. This limits the performance of DNA quantification, especially low experiment reproducibility. This study discusses the concept of a chip-oriented fluorimeter design. Using the analytical model, the current study analyzes the sensitivity and dynamic range of the fluorimeter to fit the requirements for detecting fluorescence in nano-liter volumes. Through the optimized processes, a real-time PCR on a chip system with only one nano-liter volume test sample is as sensitive as the commercial real-time PCR machine using the sample with twenty micro-liter volumes. The signal to noise (S/N) ratio of a chip system for DNA quantification with hepatitis B virus (HBV) plasmid samples is 3 dB higher. DNA quantification by the miniature chip shows higher reproducibility compared to the commercial machine with respect to samples of initial concentrations from 10^3^ to 10^5^ copies per reaction.

## 1. Introduction

Mullis and coworkers [1] first introduced the polymerase chain reaction (PCR) in 1986 to replicate specified DNA fragments *in vitro* by thermal cycling. A basic PCR run can be broken up into three phases: exponential amplification with exact doubling of the product accumulated at every thermal cycle; the linear stage as the reaction is slowing; and the plateau at the end, as the reaction components are consumed and products begin to degrade. [2] Traditional analysis methods use agarose gel electrophoresis to obtain DNA quantification results by molecular size discrimination. Detecting DNA amplification occurs at the final phase of the PCR. Analysis work takes a long time and the quantification results may not be precise due to the products’ degradation.

Developing micro-electro-mechanical-systems (MEMS) technology has recently evolved from a real-time PCR machine to a lab-on-chip system. Researchers have extensively studied miniaturizing a biological instrument and have constructed and tested several prototypes. This study collected published papers, investigated the prototype design of each chip, and compared lab-on-chip system specifications with commercial machines to determine the advantages of a real-time PCR.

Real-time PCR on a chip consists of a miniature chip and a fluorescence detection system. Thermal cycling control of the PCR is enabled by the heater and sensor, integrated on the chip. Literature in 2004 reported a miniaturized, fully-automated, PCR-based detection system for rapid detection of bacteria [3]. Using an automated detection platform with an integrated microprocessor, pumps, valves, thermal cycler, and fluorescence detection modules, microchips are used to purify and detect bacterial DNA by real-time PCR techniques. The Bio Lab of the Samsung Advanced Institute of Technology [4] reported a rapid real-time micro-scale chip-based PCR system and demonstrated successful screening of HBV-infected patients. A research institute in Singapore [5] that proposed a virtual reaction chamber (VRC) reported a lab-on-chip system at the same year. The VRC chip, which is a water-based droplet enclosed by oil and placed on a heater, only requires half a sec to heat from 72 °C to 94 °C, and two sec to cool from 94 to 55 °C, corresponding to a cooling rate of 20 °C/sec. Beer *et al*. [6] in 2007, proposed applying digital microfluidics to real-time PCR, which combine the on-chip processing of pico-liter samples for establishing a real-time PCR assay. This was the first lab-on-chip system for pico-liter droplet generation and PCR amplification with real-time fluorescence. Their work demonstrated a six-order magnitude reactor size reduction from commercial real-time PCR systems. In 2009, a nano-liter volume droplet PCR was proposed again for real-time analysis. Low-power (∼30 mW) laser radiation was employed as an optical heating source for a high-speed PCR, enabling DNA amplification in nano-liter droplets dispersed in an oil phase [7], which provides fast heating and completion of the forty cycles of PCR in 370 s. The assay performance was quantitative and its amplification efficiency was comparable to that of a commercial instrument. Taqman probes provide real-time readouts of the nano-liter droplets on the chip.

Although researches have rapidly developed miniature devices and extremely low PCR reaction volume, most chip prototypes still employ commercially available fluorescence detectors. The optical systems are very similar to those of commercial instruments, except for some compact designs, which use fibers as coupling components [8]. From 2000 onwards, little research can be found focusing on high sensitivity and high optical resolution fluorescent detection systems to specifically improve the performance of biochip DNA quantification, except for Ruckstuhl [9]. Instead of fluorescence detection, a chip prototype uses electrochemical detection. However, the presented sensitivity is not comparable with fluorescence detection [10]. To summarize, the detection system developed for the real-time PCR on a chip focuses mainly on miniaturization, with little progress in improving sensitivity and experiment reproducibility, which are both critical to biomedical instruments.

This study promotes the concept of a chip-oriented fluorimeter design. Using the analytical model, this work analyzes the sensitivity and dynamic range of the fluorimeter to fit the requirements for detecting fluorescence in nano-liter volumes. The optimized processes not only focus on increasing the sensitivity but also aim to make the real-time PCR on a chip system reliable for DNA quantification. This research constructed a real-time PCR on a chip system with the optimized fluorescence detection system to perform DNA quantification using nano-liter volume sample. The quantification results were compared with those obtained by a commercial real-time PCR machine to verify effectiveness of the chip-oriented fluorimeter design.

## 2. Chip-Oriented Fluorimeter Design

**Figure 1.**
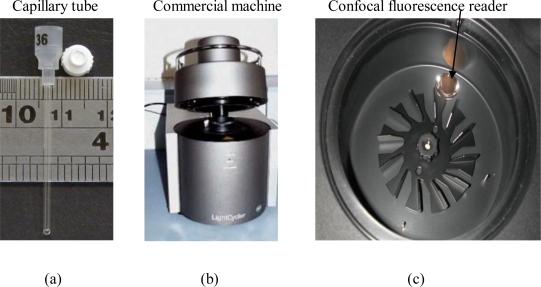

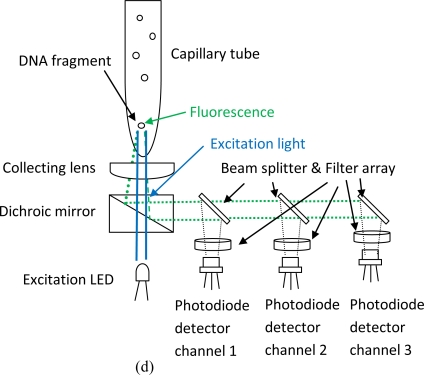
Schematic view of the fluorescence sensing system of the commercial real-time PCR machine.

A chip-oriented design starts with analysis of the fluorescence detection system employed by the commercial instrument. The real-time PCR machine from Roche employs the capillary tube as the PCR mixture vessel, shown in Figure 1(a). The tube is inserted into a thermal cycling chamber to perform PCR, and DNA fragments are amplified in vitro for quantification analysis. Figure 1(a) shows the picture of a commercial real-time PCR machine, and the arrow line indicates the insertion locations of the capillary tube.

Inside the machine, a confocal fluorimeter is equipped for fluorescence detection. The mirror shown on Figure 1(c) is the aspheric lens for collecting fluorescence. Figure 1(d) shows the scheme of the confocal fluorescence reader. The excitation light beam from an LED is focused onto the capillary tube through the dichroic mirror and aspheric lens. The dichroic mirror indicated in Figure 1(d) enables the excitation light to go directly through the aspheric lens while also allowing the fluorescence from the PCR mixture to be reflected back to the detection module. Figure 1 shows that the dichroic mirror plays a key role for confocal fluorescence detection since it allows both excitation and fluorescence detection to share the same focusing path but in inversed directions. This design is suitable for application to a real-time PCR machine since only one connection point is needed to connect to the thermal cycler and therefore less interference is encountered.

**Figure 2.**
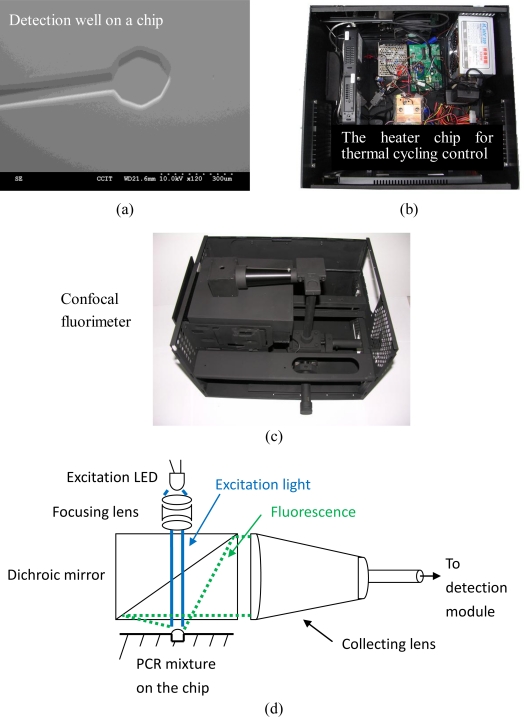
Schematic view of the confocal fluorimeter design for real-time PCR on a chip.

Confocal fluorescence detection can also work well for the real-time PCR chip as it is straightforward and inexpensive. A major benefit is the redundancy of a dynamic focus control. This study proposes developing a confocal fluorescence reader for sensitive detection with the nano-liter volume sample. Figure 2 shows the proposed design of the confocal fluorimeter for real-time PCR on a chip.

The planarity of the chip causes the focusing problem and can be solved by a suitable focusing lens arrangement as reported in the reference [9]. The other problem is the excitation and fluorescence collection in the micro-scale, and the topic to be solved in this study. As shown in Figure 2(a), a nano-liter volume sample is loaded into the circular hole with a scale of 300 μm. The whole chip is assembled onto a micro heater for performing PCR, as shown in Figure 2(b).

The chip and the heater are integrated in a box. The fluorescence detector is on the top and aligned to the detection point on the chip, as shown in Figure 2(c). The scheme of the confocal fluorescence detector for a real-time PCR chip is shown in Figure 2(d). Apart from being on top, the fluorimeter is similar to the one fitted for a machine. As mentioned above, the excitation light is focused onto the detection point on the chip by the focusing lens; the dichroic mirror splits the excitation light and fluorescence. Fluorescence is collected by the aspheric (collecting) lens and then focused onto the detection module consisting of different photodiode detector channels.

As indicated in the previous section, the chip’s problematic fluorimeter design is not only a matter of focusing; the sensitivity and dynamic range of the detector should also be well determined for detecting fluorescence effectively. This work proposes an analytical model, discussed as the following.

The following equation [11] analyzes a general expression for emissive fluorescence from a PCR mixture:

(1) $$\Delta F=I_{\lambda }(0)\times \int_{0}^{L}\int_{0}^{2\pi }\int_{\varphi_{1}(x,\theta ,\varphi )}^{\varphi_{2}(x,\theta ,\varphi )}\text{sin}\varphi \sum_{i=1}^{N}\phi_{i}\cdot a_{i,\lambda }\cdot c_{i}\left(\frac{I_{\lambda }\;(x)}{I_{\lambda }\;(0)}\right)\left(\frac{I_{\lambda^{\prime }}\;(L)}{I_{\lambda^{\prime }}\;(0)}\right)d\varphi d\theta \mathrm{dx}$$

where ΔF denotes fluorescence signal intensity that is proportional to multiplication of *I*_λ_(0) excitation light intensity and labeling dye quantum yield *ϕ_i_*, *a*_*i*,λ_ denotes the molar extinction coefficient, and *c_i_* denotes DNA template concentration at the ith PCR thermal cycle. Two fractional terms:

$$\left(\frac{I_{\lambda }\;(x)}{I_{\lambda }\;(0)}\right)\;\text{And}\;\left(\frac{I_{\lambda^{\prime }}\;(L)}{I_{\lambda^{\prime }}\;(0)}\right)$$

denote excitation light decay due to labeling dye absorption and emissive fluorescence decay as excitation light and emissive fluorescence pass through the DNA sample in a capillary tube to the collecting lens with a length *L*. The integration is performed along the DNA sample length, around the whole core angle *θ* from 0 to 2π, and over the solid angle *φ* from *φ*_1_ too *φ*_2_. Both upper and lower limits of the solid angle notably vary with the design of the optical sensing device. The summation index, *N*, depends on the number of different DNA labeling dyes in the PCR mixture.

**Figure 3.**
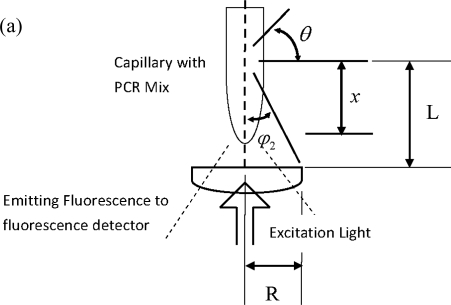

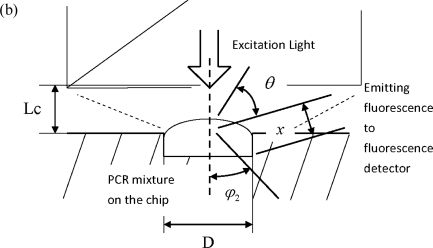
Definitions of the core angle, the solid angle and the integration length for the commercial RT-PCR machine with a con-focal excitation.

Figure 3(a) shows the definition of the core angle, solid angle, and sample length of the fluorimeter of the commercial real-time PCR machine. According to the schematic drawing, the core angle, *φ*, ranges from 0 to tan^−1^(R/*x*) in which R is the radius of the collecting lens, as shown in Figure 3(a).

Then, Equation (1) can be reduced to:

(2) $$\Delta F=2\pi \cdot I_{\lambda }\;(0)\int_{0}^{L}\int_{0}^{{\text{tan}}^{-1}(R/x)}\text{sin}\varphi \sum_{i=1}^{N}\phi_{i}\cdot a_{i,\lambda }\cdot c_{i}\left(\frac{I_{\lambda }\;(x)}{I_{\lambda }\;(0)}\right)\left(\frac{I_{\lambda^{\prime }}\;(L)}{I_{\lambda^{\prime }}\;(0)}\right)d\varphi^{\prime }\mathrm{dx}$$

This study only considers one dye for labeling the DNA template. The summation index, *N*, is equal to one. The decay of excitation light: $\left(\frac{I_{\lambda }\;(x)}{I_{\lambda }\;(0)}\right)$, can be estimated by the Beer-Lambert law as:

(3) $$\left(\frac{I_{\lambda }\;(x)}{I_{\lambda }\;(0)}\right)=\text{exp}\left(-x\cdot a_{i,\lambda }\cdot c_{i}\right)$$

The decay of emissive fluorescence:

$$\left(\frac{I_{\lambda^{\prime }}\;(L)}{I_{\lambda^{\prime }}\;(0)}\right)$$

is affected by three factors: emissive fluorescence divergence due to the PCR mix and capillary tube wall, emissive fluorescence absorption by the DNA sample solution, and Raman’s scattering effect. The first factor, emissive fluorescence divergence, includes the effect due to refraction of the DNA sample solution and capillary tube wall. Therefore, only a fraction of emissive fluorescence travels to the detector.

By considering the divergence factor alone, the decay of emissive fluorescence can be estimated by:

(4) $$\left(\frac{I_{\lambda^{\prime }}\;(L)}{I_{\lambda^{\prime }}\;(0)}\right)=\text{exp}\left(-\frac{L}{r}\right)$$

where *r* is the focusing radius of the DNA sample capillary tube, and the unit for both length parameters is mm.

By considering the absorption factor alone, the decay of emissive fluorescence can be referred to the database [12] and is given by:

(5) $$\left(\frac{I_{\lambda^{\prime }}\;(L)}{I_{\lambda^{\prime }}\;(0)}\right)=\text{exp}(0.000498\cdot L)$$

By considering Raman’s scattering effect alone, the decay of emissive fluorescence can be expressed as:

(6) $$\left(\frac{I_{\lambda^{\prime }}\;(L)}{I_{\lambda^{\prime }}\;(0)}\right)=\text{exp}\left(3\times 10^{-9}\cdot L\right)$$

with Raman’s scattering coefficient obtained from reference [12].

The coefficient in the bracket for Equation (4) is 1.2903 because the focusing radius of the DNA capillary, r is equal to 0.775 mm in this study. By comparing the coefficients in the brackets for Equations 4–6, the value for Equation (4) is much larger than those for both Equations (5) and (6). Therefore, the decay of emissive fluorescence can be rightly presented by Equation (4) alone.

By substituting Equation (4) into Equation (2), the emissive fluorescence intensity of DNA labeling dye in the PCR mix can be obtained by the expression:

(7) $$\Delta F=2\pi \cdot I_{\lambda }\;(0)\cdot \int_{0}^{L}\int_{0}^{{\text{tan}}^{-1}(R/x)}\text{sin}\varphi \cdot \phi_{i}\cdot a_{i,\lambda }\cdot c_{i}\cdot e^{-x\cdot a_{i,\lambda }\cdot c_{i}}\cdot e^{-\frac{L}{r}}d\varphi \mathrm{dx}$$

In this study, both the fluorophore quantum yield coefficient, *ϕ_i_* and the emissive fluorescence decay are constants and can be taken out from the integrand. A dimensionless signal gain can be defined as the ratio of emissive fluorescence signal: Δ*F*, to the excitation light, *I*_λ_(0). Equation (7) can be rearranged as:

(8) $$\frac{\Delta F}{I_{\lambda }\;(0)}=2\pi \cdot \phi_{i}\cdot e^{-\frac{L}{r}}\cdot \int_{0}^{L}\int_{0}^{{\text{tan}}^{-1}(R/x)}\text{sin}\phi \cdot a_{i,\lambda }\cdot c_{i}\cdot e^{-x\cdot a_{i,\lambda }\cdot c_{i}}\;d\varphi \mathrm{dx}$$

The inner integral can be expressed as the following:

(9) $$\int_{0}^{{\text{tan}}^{-1}(R/x)}\text{sin}\varphi^{\prime }\cdots d\varphi^{\prime }=\left(1-\frac{x}{\sqrt{x^{2}+R^{2}}}\right)$$

By substituting Equation (9) into Equation (8), it yields:

(10) $$\frac{\Delta F}{I_{\lambda }\;(0)}=2\pi \cdot \phi_{i}\cdot e^{-\frac{L}{r}}\cdot \int_{0}^{L}a_{i,\lambda }\cdot c_{i}\cdot \left(1-\frac{x}{\sqrt{x^{2}+R^{2}}}\right)e^{-x\cdot a_{i,\lambda }\cdot c_{i}}\mathrm{dx}$$

A transition parameter S with a unit of cm^−1^ is given by:

(11) $$S=-\left\{\frac{\text{log}\left(1-\frac{x}{\sqrt{x^{2}+R^{2}}}\right)}{x}\right\}$$

Equation (10) becomes:

(12) $$\frac{\Delta F}{I_{\lambda }\;(0)}=2\pi \cdot \phi_{i}\cdot e^{-\frac{L}{r}}\int_{0}^{L}a_{i,\lambda }\cdot c_{i}\cdot e^{-x\cdot \left(a_{i,\lambda }\cdot c_{i}+S\right)}\mathrm{dx}$$

This study treats S as a constant. The value of the integral can be obtained as:

(13) $$\frac{\Delta F}{I_{\lambda }\;(0)}=\frac{2\pi \cdot \phi_{i}\cdot e^{{\frac{L}{r}}_{2}}\cdot a_{i,\lambda }\cdot c_{i}\cdot \left[-e^{-L\left(a_{i,\lambda }\cdot c_{i}+S\right)}+1\right]}{a_{i,\lambda }\cdot c_{i}+S}$$

From Equation (13), the emissive fluorescence intensity at every PCR cycle performed on the machine can be predicted.

Figure 3(b) shows the definitions of core angle, solid angle, and integral length of the chip prototype, different from the machine. The decay of excitation light *I*_λ_(*x*)/*I*_λ_(0) equals 1, due to planar focusing. The emissive fluorescence decay should be revised as a point light source since fluorescence comes from the micro-scale detection well. The following equation is used to simulate the point light source decay:

(14) $$\left(\frac{I_{\lambda^{\prime }}\;(Lc)}{I_{\lambda^{\prime }}(0)}\right)=\frac{1}{4\pi {\left(\mathrm{Lc}/D\right)}^{2}}$$

where D is the diameter of the detection well and Lc is the distance between the detection well on the chip and the bottom of the dichroic mirror.

Substituting Equation (14) into Equation (2), it becomes:

(15) $$\Delta F=2\pi \cdot I_{\lambda }(0)\cdot \int_{0}^{x}\int_{0}^{\pi /12}\text{sin}\varphi^{\prime }\cdot \phi_{i}\cdot a_{i,\lambda }\cdot c_{i}\cdot \frac{1}{4\pi {\left(\mathrm{Lc}/D\right)}^{2}}d\varphi^{\prime }\mathrm{dx}$$

The core angle *φ*′ is confined by *L* and *D*, as shown in Figure 3(b). Since detection on the chip employs one microscope cover slip to prevent contaminations, its thickness determines the value of *L* and thus the value of the solid angle can be obtained as π /12.

The integral can easily be derived as:

(16) $$\frac{\Delta F}{I_{\lambda }(0)}=0.017\cdot \frac{1}{{\left(\mathrm{Lc}/D\right)}^{2}}\cdot x\cdot \phi_{i}\cdot a_{i,\lambda }\cdot c_{i}$$

Equation (16) can be used to predict the emissive fluorescence intensity at each PCR cycle performed on the chip. Using simulation results, the appropriate sensitivity and dynamic range of fluorescence detection system for a chip can be chosen. The so-called ‘Chip-Oriented Fluorimeter Design’ is in accordance with fluorescence changes in brightness. Furthermore, from Equation (13) and Equation (16), one can compare the different fluorescence increment trends of the machine with that on the chip. Equation (16) shows the direct correlation of fluorescence intensity and the DNA template concentration but Equation (13) gives the complicated iteration of concentrations to determine the intensity. This predicts that the fluorescence detection on a chip can have higher sensitivity than the machine.

## 3. Experimental Apparatus

To verify the above hypothesis and concepts of the chip-orientated fluorimeter design, the current study constructed real-time PCR on a chip system to perform fluorescence detection and DNA quantification in the nano-liter volume and arranged related experiments. Experiments include the sensitivity test and DNA quantification experiments.

### 3.1. Chip with Nano-Liter Volume Detection Well

**Figure 4.**
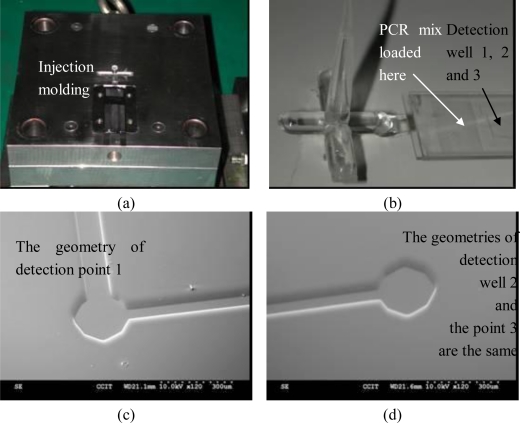
SEM pictures of detection well geometries.

To obtain a transparent chip with precise geometry, especially the nano-liter volume well, this study employed a SUMITOMO SE30S all electric injection molding machine for injection fabrication of Polymethyl methacrylate (PMMA) material replicas. The mold insert was made of wet etching silicon. The assembly condition of the insert and mold base can be referred to in Figure 4(a). Since the injection parts have a micro-scale structure, the weld line should be avoided. Subsequently the single end gated fill is the only solution for the mold design. The injection pressure was set at 1,000 kgf/cm^2^ and the filling pressure was set at 800 kgf/cm^2^. The injection speed of the filling stage was 80 mm/s and the speed was slowed down to 50 mm/sec during the packing stage. The injection temperature of PMMA was 320 °C and the mold was kept at 90 °C by oil circulation. Before part ejection formation, the mold was cooled down to room temperature in 10 s.

Figure 4(b) refers to the formation overview. One piece of chip is fabricated in an injection process. A long micro channel is distributed on the chip and three branches at the end of the channel. The detection wells for fluorescence detection are connected to each branch. Figures 4c,d refer to the SEM pictures of Well 1 and Well 2 and Well 3 has the same geometry as Well 2. The diameter of the detection well is 160 μm and the depth is 50 μm. The total volume is exactly 1 nano-liter. Since it is difficult to dispense with one nano-liter in volume, the chip employs the micro-channel to deliver the PCR mixture from the meanders to the detection wells by capillary force. A 10 μL PCR mixture is dispensed through the meanders of the micro-channel, and then the chip is covered by a microscope cover slip for fluorescence detection.

Since the PCR mixture may penetrate between the chip and the cover slip, the operator must visually inspect the chip and check if any penetration occurs and influences detection. Fluorescence detection should be carried out at each detection point. Three detection points should have similar fluorescence readings with less than 3% deviation. This helps confirm that the nano-liter volume detection wells are fulfilling their purpose sufficiently and obtains the correct fluorescence readings.

The chip made by PMMA injection is only the vessel for the PCR mixture, referred to in the following section as the ‘PCR Vessel Chip’. To perform real-time PCR, a fluorimeter and the heater chip for thermal cycling control are integrated with the PCR vessel chip.

### 3.2. Real-Time PCR on a Chip System Integration

**Figure 5.**
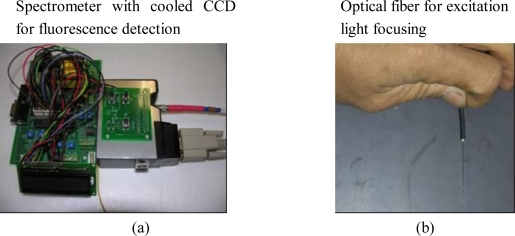

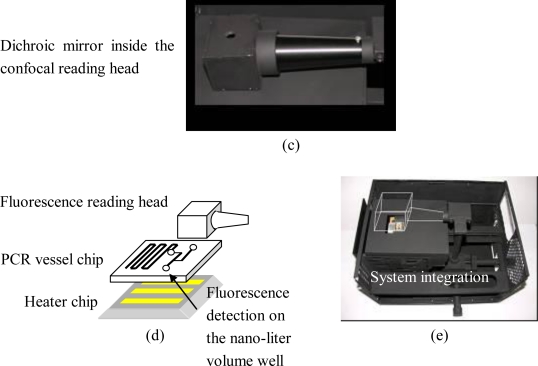
System integration of the PCR vessel chip, heater chip and fluorescence detector.

As shown in Figure 5 (a), a spectrometer, with cooled Charge-Coupled Devices (CCD), was applied to the chip-oriented fluorimeter design. The spectrometer was used for several reasons, the first being that it has an optical grating that provides sub-channel detection features similar to photodiode arrays, and the second, that it provides continuous band wavelength detection capabilities; the cooled CCD generates lower noise and therefore provides higher sensitivity. Combined with the spectrometer, the two can achieve an even lower sensitivity while also providing higher dynamic range detection capabilities, which are the two most important parameters for the chip’s oriented design.

Figure 5 (b) shows the optical fiber used for focusing the excitation light. The focusing lens, 100 μL in size, is attached to the tip of the optic fiber and is used to focus the excitation light into the well of the chip which is 150 μm in diameter. This is inserted into the hole of the confocal reading head where fluorescence is collected as shown in Figure 5 (c). The PCR mixture is loaded in the micro-scale well of the vessel chip and the PCR vessel chip is attached above the heater chip for thermal cycling control. Placed above the PCR vessel chip is the fluorescent detection head. Figure 5(d) presents the schematic view of the PCR vessel chip, heater chip, and fluorimeter assembly combining both the vessel chip and the heater chip, DNA fragments can be amplified in vitro. Using the fluorimeter, fluorescence from the micro-well is recorded for DNA quantification. The real-time PCR chip system is now complete and the fluorimeter should be optimized according to the geometry of the PCR vessel chip - the so-called chip-orientated fluorimeter design.

For thermal cycling control, the current study employed a Microprocessor Unit (MCU) (ATMEL, 89c2501, USA) to control the power input to the heater chip based on a PI control algorithm. The surface temperature can be maintained precisely with a variation of ±0.6 °C at 95 °C, a variation of ±0.4 °C at 53 °C, and a variation of ±0.6 °C at 72 °C without any significant overshooting or undershooting.

### 3.3. Commercial Real-Time PCR Machine

This research tested the commercial product (LightCycler ® Systems for Real-Time PCR, Roche) for comparative experiments. The Light-Cycler System is among the most widely utilized real-time amplification system available, with approximately 6,600 instruments in the global market [13]. Chapter 2 previously introduced the fluorescence detection system design with three discrete fluorescence detection channels. The test DNA sample was loaded into a 20 microliter capillary tube and sealed with a plastic plug. The capillary tube has an inner diameter of 0.8 mm and an outer diameter of 1.15 mm. The total reaction volume is 20 microliters.

### 3.3. Fluorimeter Sensitivity Test

The fluorimeter sensitivity is illustrated by detecting fluorescein dye which is used for calibrating the fluorescence detection system [14]. The commercial machine is first tested using serial diluted fluorescent reagent samples (10,000, 1,000, 100, 10, 1, 0.1, 0.01 fmol) with negative control (water) as the background value. Fluorimeter sensitivity of the commercial machine is analyzed and then used as the baseline for comparing the fluorimeter for the chip. To verify chip sensitivity, a similar test is carried out using the same serial diluted fluorescent reagent samples. The serial diluted sample can also be used to test the dynamic range of the fluorescence detection system. However, a DNA quantification experiment is still needed to verify that the fluorimeter is optimized for the chip.

### 3.4. DNA Quantification Experiments

To verify the on-board chip fluorescent design, this study performed DNA quantification experiments on quantitative measurements of hepatitis B virus (HBV) concentration on Standard Calibrator (SC-11), for a DNA fragment. The initial DNA copy in the PCR mixture ranged from 10 to 10^6^ copies per reaction. A Light Cycler-Fast Start DNA master, LC Red-640 labeling dye and Fluorescein dye for internal control was employed as the PCR mixture in all runs. The thermal cycling condition is stated as follows: the condition requires 10 min incubation at 95 °C. After incubation, each thermal cycle undergoes 3 s of 95 °C denaturation, 10 sec of 53 °C annealing, and 16 s of 72 °C elongation, to amplify the DNA fragment.

DNA amplification on the PCR vessel chip is performed and the fluorescence is captured by the fluorimeter design for the chip. The fluorescence readings can be used to quantify DNA samples with copy numbers ranging from 10 to 10^6^ copies per reaction. DNA quantification results are compared with those obtained with the commercial machine. The comparison results can be used to illustrate that the fluorimeter is optimized for the fluorescence detection on the chip with only a nano-liter volume of PCR mixture.

As discussed in Chapter 1, the main purpose of this study is to increase reliability of DNA quantification experiments performed on a chip. The 5-time average of measurement results (Intra-assay) with the same experiment setup was used to determine reproducibility of DNA quantification. This study used coefficients of variations (CV) to express the uncertainty of DNA quantification. The CV with percentage unit is defined as:

(17) $$\mathrm{CV}=-\frac{{\text{log}}_{10}\left(\frac{1}{n}\sum_{n}{\mathrm{ui}}_{M}\right)-{\text{log}}_{10}\left({\mathrm{ui}}_{s}\right)}{{\text{log}}_{10}\left({\mathrm{ui}}_{s}\right)}\times 100\%$$

where *ui* denotes the initial number of copies of the test sample measures on a chip, suffix M denotes the measured results of the amount of DNA fragments in the test sample; the suffix *S* denotes the known correct DNA amount from standard samples, collection of n-times measurements, and confirming correct values. CV values are used to evaluate the reproducibility of DNA quantification experiments. High CV value denotes large deviation in each experiment and can be considered as low reproducibility.

## 4. Results and Discussion

**Figure 6.**
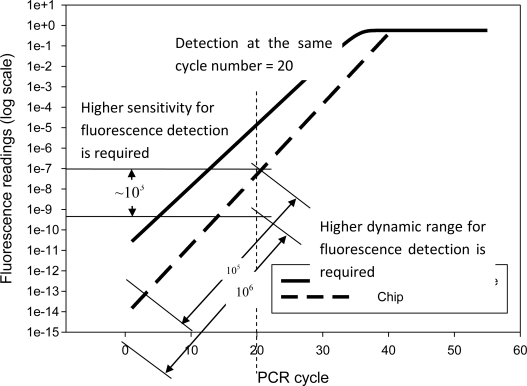
Simulated fluorescent intensities of chip and commercial machine with respect to PCR cycles.

Using the analytical model discussed in Chapter 2, this work simulated fluorescence increment, with respect to each PCR cycle, as shown in Figure 6. This simulation is based on a DNA sample of 10^6^ initial copies per reaction. However, the simulation results can be fit to other cases of low initial copies number. Assuming the detection point is at the 20^th^ cycle, fluorimeter sensitivity can be determined by different fluorescent intensities between the machine and the chip, illustrated by the curve in Figure 6. Observations at the 20^th^ PCR cycle show that the chip requires a much higher sensitivity, around 10^3^ times greater than that of the machine, and a higher dynamic range, ten times greater for fluorescence detection. These results derive from analysis of the chip-oriented fluorimeter design and thus propose the fundamental requirements.

Referring to the user manual [14], the commercial Light Cycler ® Systems used the Photo hybrid as the detector. Its sensitivity at 530 nm, using 20 μL sample volume, is 15 fmol fluorescein, and the dynamic range of detection sensitivity can be adjusted by the factor of 1 to 256 (e.g., the dynamic range of the machine is 256). Since the sensitivity requirement is one thousand times higher than the commercial machine, detecting 0.015 fmol fluorescein is needed. Since the dynamic range should be higher than the commercial by 10 times, the dynamic range must be set to 2,560. Therefore, the detection sensitivity and dynamic range of the fluorimeter on the chip is set according to these quantitative values.

**Figure 7.**
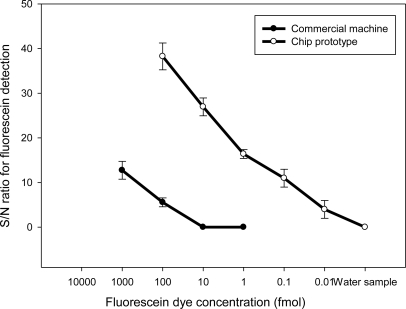
Signal to noise (S/N) ratio of the standard sample with fluorescein dye illustrates that the fluorimeter design for the chip has larger dynamic range and higher sensitivity than the machine. The commercial machine uses the sample of 20 microliter volume and the sample volume on a chip is only 1 nano-liter.

This research carried out the fluorescein sensitivity test to verify that the chip prototype can achieve the required sensitivity and dynamic range setting. Using spectra of the serial diluted fluorescent reagent samples and the negative control sample, the results illustrate successful detection from 100 fmol fluorescent reagent samples to 0.01 fmol. The dynamic range is 100/0.01 = 10,000, which exceeds the requirement of 2,560. Figure 7 shows that fluorescein detection on the chip has a ratio of 3 dB for detecting a 0.01 fmol sample, but the machine can only effectively distinguish high concentration samples from 1,000 to 10 fmol. Thus, chip prototype sensitivity is proven to be 1000 times higher than the commercial machine, fulfilling all design requirements of the chip-oriented fluorimeter. Such high sensitivity and large dynamic range are achieved for a small quantity sample with only 1 nano-liter of volume.

Although the desired requirement can be satisfied, DNA quantification experiments still need to be carried out to verify that the fluorimeter is really optimal for the chip and can achieve successful DNA quantification. The homemade spectrometer provides continuous wavelength detection from 400 to 800 nm. The fluorescence increment can be read out around the central wavelength at 640 nm that corresponds to the peak of LC-640 dye. Except for the increment, fluorescence of the internal control using Fluorescien dye is monitored at the same time. That signal is used to calibrate the fluorescence increment signal to obtain accurate quantitative results. The details about fluorescence signal processing and how to obtain quantification results from the fluorescence increment can be referred to in the author’s previous work [15].

**Figure 8.**
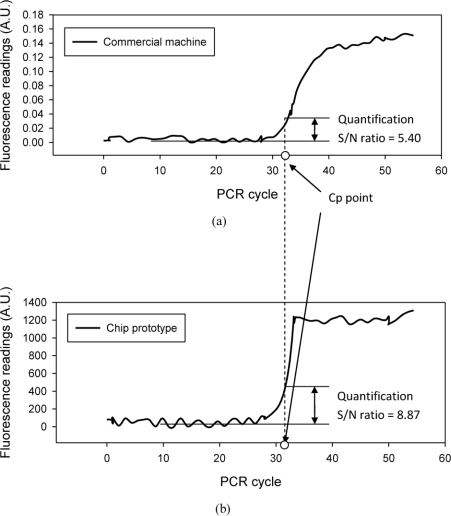
Fluorescence increment curves obtained by the commercial machine (a) and the chip (b) from the sample of 10^5^ initial copies per reaction. The figures illustrate that the chip-orientated fluorimeter design provides a higher S/N ratio than the commercial machine at the same detection point, Cp. The higher S/N ratio indicates achieving more sensitive detection in the 1 nano-liter volume by the fluorimeter.

This study compared DNA quantification results obtained from the chip prototype with the commercial machine. Figure 8 (a) and (b) show the fluorescence increment curves obtained from both the chip and machine. Both curves are the sample quantification with 10^5^ copies per reaction. The commercial machine determines the detection point at the 32^nd^ cycle, which is applied to analysis of the fluorescence curve obtained on the chip. At the same cycle point, the fluorescence curve of the chip prototype has a 3.47 dB higher S/N ratio than the machine. The high S/N ratio denotes that the signal can be determined with less noise interference or the detection point can reach to the 30^th^ cycle to obtain quantification results during the early phase of PCR. These results indicate that the chip-oriented fluorimeter design increases reproducibility of DNA quantification since it provides a higher S/N ratio than the commercial machine.

**Figure 9.**
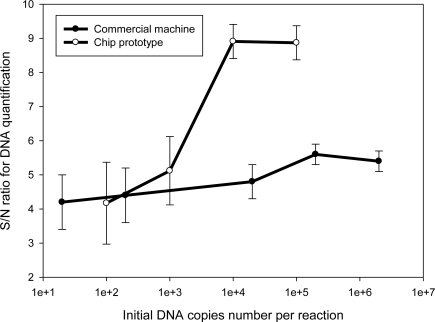
S/N ratios of the commercial machine and the chip for samples of serial concentration levels from 10^6^ to 10 copies per reaction. The comparison is used to verify performance of the real-time PCR chip system and the results show that the chip does not work as well as the machine for analyzing the sample that contains lower initial copy numbers than 10^3^ copies per reaction.

Quantification analysis of the samples with serial initial copy numbers shows a notable result. Figure 9 show that the fluorimeter provides high S/N ratios for quantification analysis for samples with a high number of initial DNA copies. However, the S/N ratio drops when the test sample contains low numbers of copies of 10^3^ per reaction. Regarding the low copy number of 10^2^, the compared S/N ratio is even lower than the machine’s. Since the sample only contains ten copies, the real-time PCR chip system is unable to amplify DNA and cannot obtain and results. These results illustrate that the real-time PCR chip cannot provide high S/N ratios for analyzing samples with low initial copy numbers.

**Figure 10.**
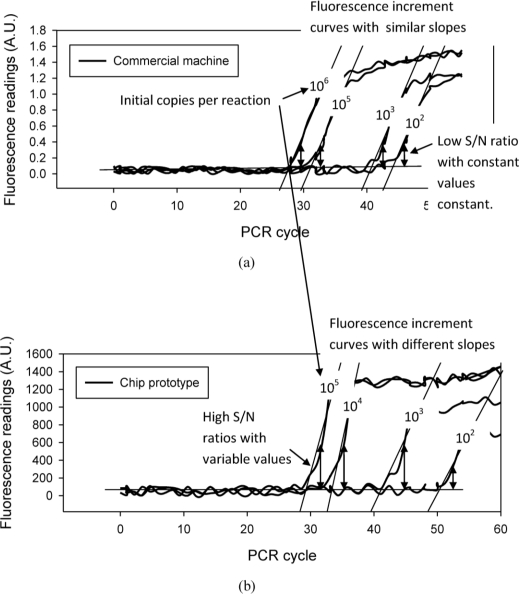
Quantification results of a commercial machine (a) and the chip (b) for a serial sample of different initial copy numbers. The figures illustrate that the chip-orientated fluorimeter design provides high sensitive detection but the fluorescence increment curves with different slopes yield variable S/N ratios. This indicates the inconsistency and low reproducibility of DNA quantification by a chip with only one nano-liter sample volume.

To find out the reason, this work examines fluorescence increment curves for a serial sample. As shown in Figure 10(a), the real-time PCR machine provides low S/N ratios and the values are almost a constant. Figure 10(b) gives the high S/N ratios for the chip system, but the values become increasingly lower for the sample with low initial copy numbers. This is because variable DNA amplification efficiencies cause different slopes, a known effect related to the nonspecific adsorption of biological samples to the vessel surfaces. The effect becomes significant as a result of the increased surface-to-volume ratio upon miniaturization, which may inhibit PCR amplification [16], thus yielding fluorescent curve variations. If only considering the fluorimeter, the results of high S/N ratios for DNA quantification analysis of the samples with high initial copy numbers still illustrate good fluorescence detection performance, especially for obtaining a signal from the nano-liter volume well; the S/N ratio can be higher than the machine’s which uses a much larger volume up to 20 microliters.

**Figure 11.**
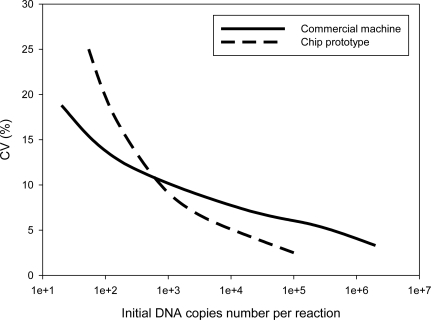
Index of experiment reproducibility by the coefficient of variant, CV (%). DNA quantification experiments using the commercial machine and the chip are compared with samples of initial DNA copy numbers from 10 to 10^6^. The results show that DNA quantification by a chip has high reproducibility for samples with high concentration levels from 10^5^ to 10^3^ copies per reaction.

Although fluorescence detection on the chip shows a higher S/N ratio than the machine, the current study carried out quantification experiments for the samples of serial concentration levels from 10 to 10^6^ copies per reaction to verify performance of a real-time PCR chip system. Due to the nano-liter volume, the maximum concentration tested on a chip was 10^5^ copies per reaction. Only fifty copies per reaction were successfully detected by the chip, due to the surface adsorption phenomena previously illustrated. Figure 11 shows the index results of experiment reproducibility.

This study carried out five inter-assay DNA quantification experiments to obtain the coefficient of variation (CV) for quantification results. The last section of Chapter 3 discusses the CV definition. CV is the index of the experiment’s reproducibility. High S/N ratio for DNA quantification denotes reliable results and thus a low CV. The results in Figure 11 correspond well to the S/N ratios previously reported and the chip obtains a high CV by the chip for samples with high concentration levels from 10^3^ to 10^5^ copies per reaction.

## 5. Conclusions

This study proposes the concept of chip-oriented fluorimeter design and analyzes a suitable sensitivity and dynamic range of the detector for fluorescence detection in the nano-liter volume well using an analytic model. With proper fluorimeter settings, the presented chip prototype demonstrates high sensitivity for fluorescence detection and a high S/N ratio for DNA quantification. Comparing commercial real-time PCR machines that use 20 micro-liter volume test samples, a chip as small as one nano-liter in volume is as much as one thousand times more sensitive. This yields better reproducibility of DNA quantification with the HBV plasmid samples of 10^3^∼10^5^ initial copy numbers, and provides results, which illustrate that the chip orientated fluorimeter design can be effectively used to improve DNA quantification performance. Due to surface adsorption effect, the chip system does not perform well for amplifying and detecting samples of low initial copy numbers. The detection limit is only fifty copies per reaction. The inhibition effect can be solved by changing the recipe of the PCR mixture or surface coating on the sample well of the chip. The reviewed literature demonstrates single copy detection on a chip [6,7]. However, most papers related to real-time PCR on a chip report lower experiment reproducibility than the machine [7,8,10,11,15], which may prevent applications of the lab-on-a-chip system. This study focuses on the fluorimeter designs for a miniature chip. Using an optimized design, this research achieves high reliable DNA quantification by the lab-on-a-chip system with extreme low sample volume to a nano-liter scale. Future development of a real-time PCR on a chip will be critical.
